# Competing risk of the specific mortality among Asian-American patients with prostate cancer: a surveillance, epidemiology, and end results analysis

**DOI:** 10.1186/s12894-022-00992-y

**Published:** 2022-03-24

**Authors:** Di Wu, Yaming Yang, Mingjuan Jiang, Ruizhi Yao

**Affiliations:** 1 Department of Urology, The First Affiliated Hospital of Guangzhou University of Traditional Chinese Medicine, No. 16 Jichang Road, Baiyun District, Guangzhou, 510405 Guangdong People’s Republic of China; 2 Department of Urology, The First Clinical Medical College of Guangzhou University of Traditional Chinese Medicine, Guangzhou, 510405 Guangdong People’s Republic of China

**Keywords:** Competing-risk, Asian-American, PCa-specific mortality, SEER

## Abstract

**Background:**

Adopted the competing-risk model to investigate the relevant factors affecting the prostate cancer (PCa)-specific mortality among Asian-American PCa patients based on the Surveillance, Epidemiology, and End Results (SEER) database.

**Methods:**

The information of 26,293 Asian-American patients diagnosed with PCa between 2004 and 2015 were extracted from the SEER 18 database. Subjects were divided into three groups: died of PCa, died of other causes, survival based on the outcomes at the end of 155 months’ follow-up. Multivariate analysis was performed by the Fine-gray proportional model. Meanwhile, subgroup analyses were conducted risk stratification by race and age.

**Results:**

Age ≥ 65 years [Hazard ratio (HR) = 1.509, 95% confidence interval (CI) 1.299–1.754], race (HR = 1.220, 95% CI 1.028–1.448), marital status (unmarried, single or widowed, HR = 1.264, 95% CI 1.098–1.454), tumor grade II (HR = 3.520, 95% CI 2.915–4.250), the American Joint Committee on Cancer (AJCC) stage (T3: HR = 1.597, 95% CI 1.286–1.984; T4: HR = 2.446, 95% CI 1.796–3.331; N1: HR = 1.504, 95% CI 1.176–1.924; M1: HR = 9.875, 95% CI 8.204–11.887) at diagnosis, radiotherapy (HR = 1.892, 95% CI 1.365–2.623), regional nodes positive (HR = 2.498, 95% CI 1.906–3.274) increased risk of PCa-specific mortality for Asian-American PCa patients, while surgical (HR = 0.716, 95% CI 0.586–0.874) reduced the risk.

**Conclusion:**

The study findings showed that age, race, marital status, tumor grade (II), AJCC stages (T3, T4, N1, M1) at diagnosis, radiotherapy, regional nodes positive and surgery was associated with the specific mortality of PCa patients among Asian-Americans.

## Background

Prostate cancer (PCa) is the second most common cancer and the fifth leading cause of cancer death among men worldwide [1]. The American Cancer Society pointed in 2020 that there were about 191,930 new cases and 33,330 deaths in the United States [2]. Despite the mortality of PCa has been gradually decreased in recent years, it is worth noting that racial differences still make PCa mortality vary widely among different groups in the United States [3]. Asian-Americans are considered as the most rapidly growing racial group in the United States [4, 5]. Several epidemiological studies have shown that PCa was the most common malignancy for nearly all Asian-American men, and they had a more advanced stage, higher-grade tumor than caucasians [6–9], which could produce a vital impact for the prognosis of the populations. Therefore, it is essential to focus on the risk factors of death in Asian-American PCa patients.

Previous studies have reported several risk factors of death for human PCa [10–12]. Pettersson et al. [13] used the Cox regression to investigate the association between age at diagnosis and prognosis for PCa patients, the result displayed that PCa had more aggressive and higher mortality for older men. But actually, death from PCa was only one of the death causes, and death caused by other diseases or traffic accidents would exist as well [14, 15], it must be admitted that Cox proportional hazard model tended to make the outcomes’ risk higher, causing bias [15]. A relatively important issue is to accurately determine which factors affecting the survival and prognosis of PCa patients for Asian-Americans. In recent years, there were studies pointed that compared with the Cox model, the competing-risk model could better estimate the risk of major outcomes of benefit when one or more competitive risks are existed, and evaluated the factors of prognosis by competing-risk model would be more helpful to identify the associated risk factors accurately [15–17].

To our knowledge, there are relatively few reports about risk factors of influenced survival in Asian-American PCa patients by using the competing-risk model. In consequence, our study adopted the competing-risk model to investigate the relevant factors affecting the PCa-specific mortality among Asian-American PCa patients based on the Surveillance, Epidemiology, and End Results (SEER) database, which could be expected to provide a reference for clinicians to accurately assess the factors of prognosis in Asian-American PCa patients.

## Methods

### Data source

Data was obtained from the SEER Database of National Cancer Institute (NCI), which captured information on cancer diagnosis, treatment and survival for approximately 30% of the United States population [18]. SEER database contains publicly available data, and the NCI does not need to get the approval of the Institutional Review Board to use it. The SEER database collected data about demographic characteristics, primary tumor site, tumor morphology, the American Joint Committee on Cancer (AJCC) stage, incidence rates, survival outcomes, cause of death and treatment among patients [19].

In the present study, a total of 30,861 Asian-American man patients with primary PCa from 2004 to 2015 were extracted from SEER 18 database by SEER*STAT v8.3.9, using the ICD codes of C61.9-prostate for diagnoses. We excluded 190 patients with lack of survival time and 4378 patients with unknown variables such as grade, T stage, and N stage. The final analysis included 26,293 eligible patients with PCa. Participants were divided into three groups: died of PCa, died of other causes, survival based on the outcomes at the end of 155 months’ follow-up. Not required Ethics Committee approval or Institutional Review Board approval, because of individual patient data has been removed.

### Data collection

Baseline data were collected, including year at diagnosis, age at diagnosis, marital status (married, unmarried, single, widowed), histological types (adenocarcinoma, squamous-cell carcinoma, mucinous carcinoma, sarcoma), grade (I and II), the classification of AJCC stage [20] was T (extent of tumor), N (invasion of lymph nodes), and M (presence or absence of metastasis), T stage were defined as T1 (the clinically inapparent tumor is not palpable), T2 (the tumor is palpable and confined in prostate), T3 (the extraprostatic tumor is not immobilized or does not invade adjacent structures), T4 (the tumor is immobilized or invades adjacent structures other than seminal vesicles), N stage were defined as N0 (no positive regional lymph nodes) and N1(metastases of regional lymph nodes), M stage pointed M0 (absence of distant metastasis) and M1(presence of distant metastasis), radiotherapy, chemotherapy, surgery, regional nodes positive. The situations (died of PCa, died of other causes, survival based on the outcomes at the end of 155 months’ follow-up) of the PCa patients were regarded as the outcome variables.

### Statistical analysis

Baseline data was presented by using number and percentage values. We structured competing-risk model based on the death from PCa and other causes (non-PCa). Using the univariate Gray’s test to calculate the cumulative incidence function (CIF) of interest events, and compared the cumulative incidence, screened out statistically significant variables between groups, which were included in multivariate Fine-gray proportional model for further analyze the related factors of PCa-specific mortality. Risk stratification was performed by age and race. Hazard ratio (HR) and 95% confidence interval (CI) were calculated in this study. All statistical analyses were performed using the SAS 9.4 statistical analysis software, random forest diagram was drawn by R software (version 4.20). *P* < 0.05 was considered as statistically significant.

## Results

### Baseline characteristics

The demographic and clinical characteristics of study were displayed in Table 1. 26,293 eligible Asian-American patients with primary PCa were included in our study ultimately. 1038 (3.95%) patients were died of PCa, 3619 (13.76%) patients were death from other causes, and 21,636 (82.29%) patients still survived at the end of the follow-up period (the loss of follow-up rate was 0.62% and the median survival time was 71 months). 4928 cases (18.74%) were Ethnic Chinese, other Asian-Americans (such as Japanese, Korean, Filipino, etc.) had 21,365 cases (81.26%). In this population, when they were diagnosed with PCa disease, 39.87% patients were younger than 65 years old and 60.13% patients aged ≥ 65 years. And the majority of patients has married (75.12%), 24.88% patients were unmarried, single or widowed at diagnosis. Totally 14,476 PCa patients diagnosed with grade II, 2736 (10.41%) patients at AJCC stage T3, and only 483 patients at T4 stage. 980 patients received radiotherapy, 163 patients accepted chemotherapy, surgery was the primary treatment for 10,844 (41.24%) PCa patients. Detailed baseline information was given in Table 1.

**Table 1:** Baseline characteristics of all included participants

Variables	Total (n = 26,293)	Ethnic Chinese (n = 4928)	Other Asian-Americans (n = 21,365)
Year of diagnosis, year, n (%)			
2004	2265 (8.61)	426 (8.64)	1839 (8.61)
2005	2128 (8.09)	426 (8.64)	1702 (7.97)
2006	2279 (8.67)	469 (9.52)	1810 (8.47)
2007	2524 (9.60)	517 (10.49)	2007 (9.39)
2008	2303 (8.76)	477 (9.68)	1826 (8.55)
2009	2364 (8.99)	448 (9.09)	1916 (8.97)
2010	2278 (8.66)	430 (8.73)	1848 (8.65)
2011	2367 (9.00)	412 (8.36)	1955 (9.15)
2012	1979 (7.53)	374 (7.59)	1605 (7.51)
2013	1913 (7.28)	302 (6.13)	1611 (7.54)
2014	1854 (7.05)	307 (6.23)	1547 (7.24)
2015	2039 (7.75)	340 (6.90)	1699 (7.95)
Age at diagnosis, years, n (%)			
< 65	10,484 (39.87)	1737 (35.25)	8747 (40.94)
≥ 65	15,809 (60.13)	3191 (64.75)	12,618 (59.06)
Marital status, n (%)			
Married	19,750 (75.12)	3826 (77.64)	15,924 (74.53)
Unmarried, single or widowed	6543 (24.88)	1102 (22.36)	5441 (25.47)
Histological types, n (%)			
Adenocarcinoma	25,964 (98.75)	4838 (98.17)	21,126 (98.88)
Squamous-cell carcinoma, mucinous carcinoma or sarcoma	329 (1.25)	90 (1.83)	239 (1.12)
Grade, n (%)			
I	11,817 (44.94)	2193 (44.50)	9624 (45.05)
II	14,476 (55.06)	2735 (55.50)	11,741 (54.95)
T Stage, n (%)			
T1	10,817 (41.14)	2212 (44.89)	8605 (40.28)
T2	12,257 (46.62)	2187 (44.38)	10,070 (47.13)
T3	2736 (10.41)	449 (9.11)	2287 (10.70)
T4	483 (1.84)	80 (1.62)	403 (1.89)
N Stage, n (%)			
N0	25,650 (97.55)	4824 (97.89)	20,826 (97.48)
N1	643 (2.45)	104 (2.11)	539 (2.52)
M Stage, n (%)			
M0	25,476 (96.89)	4785 (97.10)	20,691 (96.85)
M1	817 (3.11)	143 (2.90)	674 (3.15)
Radiotherapy, n (%)			
No	25,313 (96.27)	4769 (96.77)	20,544 (96.16)
Yes	980 (3.73)	159 (3.23)	821 (3.84)
Chemotherapy, n (%)			
No	26,130 (99.38)	4895 (99.33)	21,235 (99.39)
Yes	163 (0.62)	33 (0.67)	130 (0.61)
Surgery, n (%)			
No	15,449 (58.76)	3010 (61.08)	12,439 (58.22)
Yes	10,844 (41.24)	1918 (38.92)	8926 (41.78)
Regional nodes positive, n (%)			
No	5845 (22.23)	1011 (20.52)	4834 (22.63)
Yes	20,448 (77.77)	3917 (79.48)	16,531 (77.37)
Follow time (months), M (Q_1_, Q_3_)	71 (38, 107)	75 (42, 109)	71 (38, 106)
Outcomes, n (%)			
Survived at the end of the follow-up period	21,636 (82.29)	4077 (82.73)	17,559 (82.19)
Died of PCa	1038 (3.95)	171 (3.47)	867 (4.06)
Died of other causes	3619 (13.76)	680 (13.80)	2939 (13.76)

*PCa* prostate cancer

### The risk factors of the specific mortality in PCa patients by the univariate analysis

The results were showed in Table 2; when the competitive risk existed, the cumulative incidence rate of specific mortality was higher in patients who were older and had other marital status (unmarried, single or widowed), graded II, T4 stage, N1 stage, M1 stage, radiotherapy, chemotherapy, not received surgery, respectively, which were significantly difference between the groups (*P* < 0.05). Furthermore, the results of univariate analysis suggested (Table 3) that age, race, marital status, grade, T stage, N stage, M stage, radiotherapy, chemotherapy, surgery and regional nodes positive significantly affected the specific mortality of PCa (*P* < 0.05).

**Table 2:** Cumulative incidence rate of death factors in patients with prostate cancer by Gray’s test

Variables	*P*	CIF	95%CI	
			Lower	Upper
Race				
Ethnic Chinese	0.021	5.96	4.86	7.20
Other Asian-Americans		7.42	6.76	8.11
Age at diagnosis				
< 65	< 0.001	4.90	4.12	5.76
≥ 65		8.52	7.74	9.35
Marital status				
Married	< 0.001	6.67	6.02	7.35
Unmarried, single or widowed		8.72	7.47	10.08
Grade				
I	< 0.001	3.07	2.39	3.89
II		10.26	9.41	11.15
T stage				
T1	< 0.001	6.63	5.69	7.67
T2		6.00	5.27	6.80
T3		10.95	8.89	13.25
T4		37.28	30.01	44.54
N stage				
N0	< 0.001	6.48	5.91	7.08
N1		40.22	33.84	46.51
M stage				
M0	< 0.001	5.67	5.13	6.24
M1		65.17	51.15	78.37
Radiotherapy				
No	< 0.001	6.92	6.34	7.53
Yes		13.21	9.73	17.22
Chemotherapy				
No	< 0.001	7.00	6.42	7.61
Yes		31.66	21.04	42.79
Surgery				
No	< 0.001	9.01	8.17	9.91
Yes		4.25	3.67	4.89
Regional nodes positive				
No	< 0.001	3.02	2.29	3.89
Yes		8.25	7.55	8.99
Histological types				
Adenocarcinoma	0.611	7.20	6.61	7.83
Squamous-cell carcinoma, mucinous carcinoma or sarcoma		6.00	3.68	9.10

*CIF* cumulative incidence function, *CI* confidence interval

**Table 3:** Univariate analysis of the risk factors of the specific mortality in patients with prostate cancer

Variables	β	S. E	*P*	HR	95%CI	
					Lower	Upper
Age at diagnosis						
< 65				Ref		
≥ 65	0.640	0.071	< 0.001	1.897	1.650	2.180
Race						
Ethnic Chinese				Ref		
Other Asian-Americans	0.191	0.084	0.022	1.211	1.028	1.426
Marital status						
Married				Ref		
Unmarried, single or widowed	0.364	0.067	< 0.001	1.439	1.261	1.642
Grade						
I				Ref		
II	1.639	0.091	< 0.001	5.149	4.306	6.157
T stage						
T1				Ref		
T2	− 0.048	0.072	0.500	0.953	0.828	1.097
T3	0.549	0.098	< 0.001	1.731	1.429	2.096
T4	2.336	0.112	< 0.001	10.343	8.313	12.870
N stage						
N0				Ref		
N1	2.355	0.089	< 0.001	10.541	8.861	12.539
M stage						
M0				Ref		
M1	3.144	0.070	< 0.001	23.203	20.235	26.606
Radiotherapy						
No				Ref		
Yes	0.611	0.126	< 0.001	1.843	1.439	2.361
Chemotherapy						
No				Ref		
Yes	1.896	0.184	< 0.001	6.658	4.638	9.558
Surgery						
No				Ref		
Yes	− 0.734	0.072	< 0.001	0.480	0.417	0.552
Regional nodes positive						
No				Ref		
Yes	1.241	0.118	< 0.001	3.459	2.744	4.362
Histological types						
Adenocarcinoma				Ref		
Squamous-cell carcinoma, mucinous carcinoma or sarcoma	0.149	0.243	0.541	1.160	0.721	1.869

*HR* hazard ratio, *CI* confidence interval

### The risk factors of the specific mortality in PCa patients by the multivariate analysis

Variables with statistically significant differences (*P* < 0.05) were included in the competing-risk model for stepwise analysis based on the results of univariate analysis. As was depicted in Table 4, in the presence of competitive risk, patients with aged 65 years or older were more likely to have a higher risk of PCa-specific mortality compared to patients with younger than 65 years at diagnosis (HR = 1.509, 95% CI 1.299–1.754). There was a higher risk of PCa-specific mortality among other Asian-American patients (HR = 1.220, 95% CI 1.028–1.448) relative to Ethnic Chinese patients. Not only that, PCa patients who had other marital status (unmarried, single or widowed, HR = 1.264, 95% CI 1.098–1.454) also took an increased risk of the specific mortality. Similarly, patients who were grade II (HR = 3.520, 95% CI 2.915–4.250), or T3 stage (HR = 1.597, 95% CI 1.286–1.984) and T4 stage (HR = 2.446, 95% CI 1.796–3.331), or N1 stage (HR = 1.504, 95% CI 1.176–1.924), or M1 stage (HR = 9.875, 95% CI 8.204–11.887), or regional nodes positive (HR = 2.498, 95% CI 1.906–3.274), or received radiotherapy (HR = 1.892, 95% CI 1.365–2.623) were significantly associated with a rasied risk of PCa-specific mortality. Nevertheless, it’s worth noting that a reduced risk of PCa-specific mortality occurred in patients with receiving surgical (HR = 0.716, 95% CI 0.586–0.874).

**Table 4:** Multivariate analysis of the risk factors of the specific mortality in patients with prostate cancer

Variables	β	S. E	*P*	HR	95%CI	
					Lower	Upper
Age at diagnosis						
< 65				Ref		
≥ 65	0.412	0.077	< 0.001	1.509	1.299	1.754
Race						
Ethnic Chinese				Ref		
Other Asian-Americans	0.199	0.087	0.023	1.220	1.028	1.448
Marital status						
Married				Ref		
Unmarried, single or widowed	0.234	0.072	0.001	1.264	1.098	1.454
Grade						
I				Ref		
II	1.259	0.096	< 0.001	3.520	2.915	4.250
T stage						
T1				Ref		
T2	0.127	0.079	0.108	1.135	0.973	1.325
T3	0.468	0.111	< 0.001	1.597	1.286	1.984
T4	0.894	0.158	< 0.001	2.446	1.796	3.331
N stage						
N0				Ref		
N1	0.408	0.126	0.001	1.504	1.176	1.924
M stage						
M0				Ref		
M1	2.290	0.094	< 0.001	9.875	8.204	11.887
Radiotherapy						
No				Ref		
Yes	0.638	0.167	0.001	1.892	1.365	2.623
Chemotherapy						
No				Ref		
Yes	0.106	0.231	0.647	1.112	0.706	1.750
Surgery						
No				Ref		
Yes	− 0.335	0.102	0.001	0.716	0.586	0.874
Regional nodes positive						
No				Ref		
Yes	0.915	0.138	< 0.001	2.498	1.906	3.274

*HR* hazard ratio, *CI* confidence interval

### Risk stratification based on race and age

**Fig. 1:**
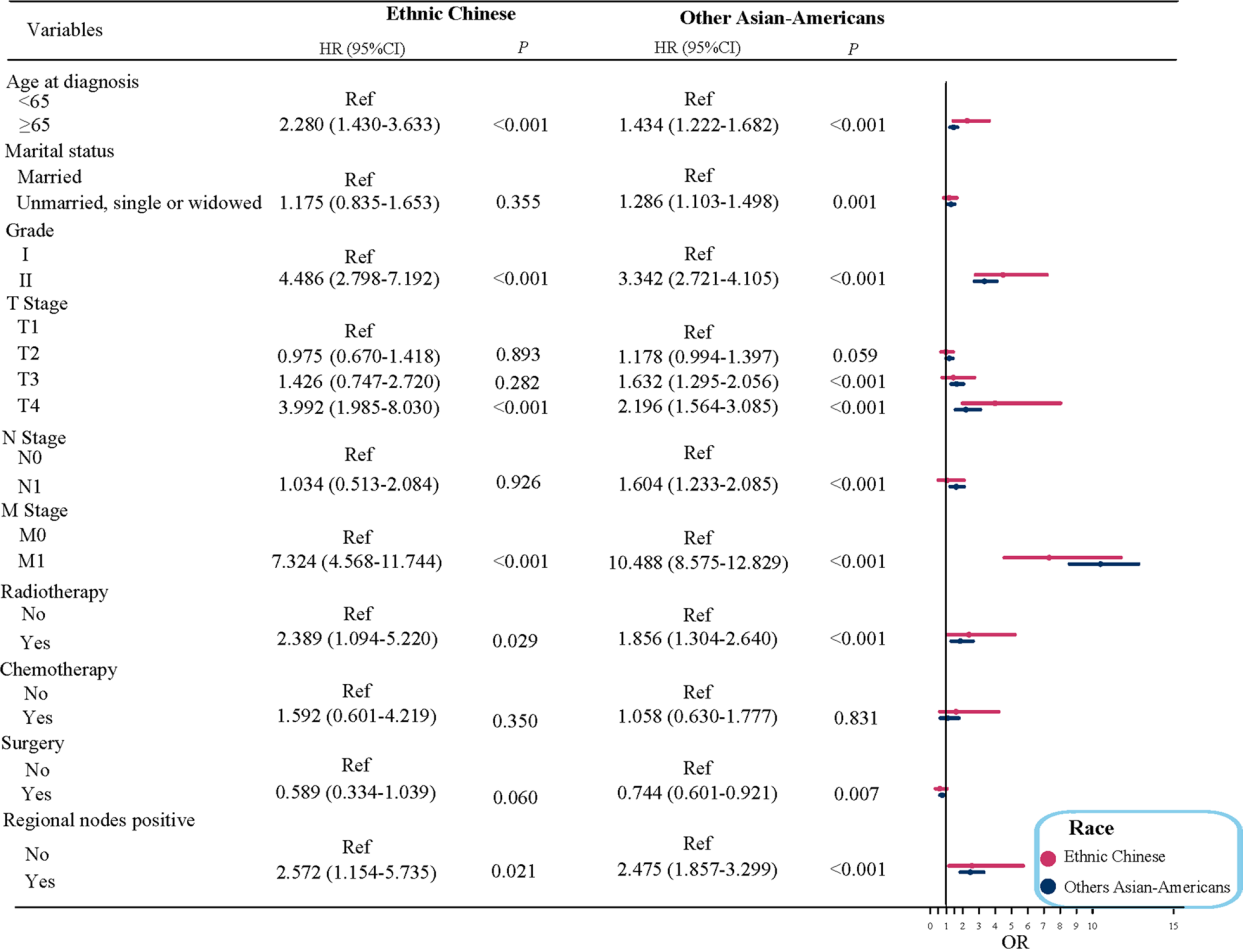
Risk stratification based on race in patients with prostate cancer

**Fig. 2:**
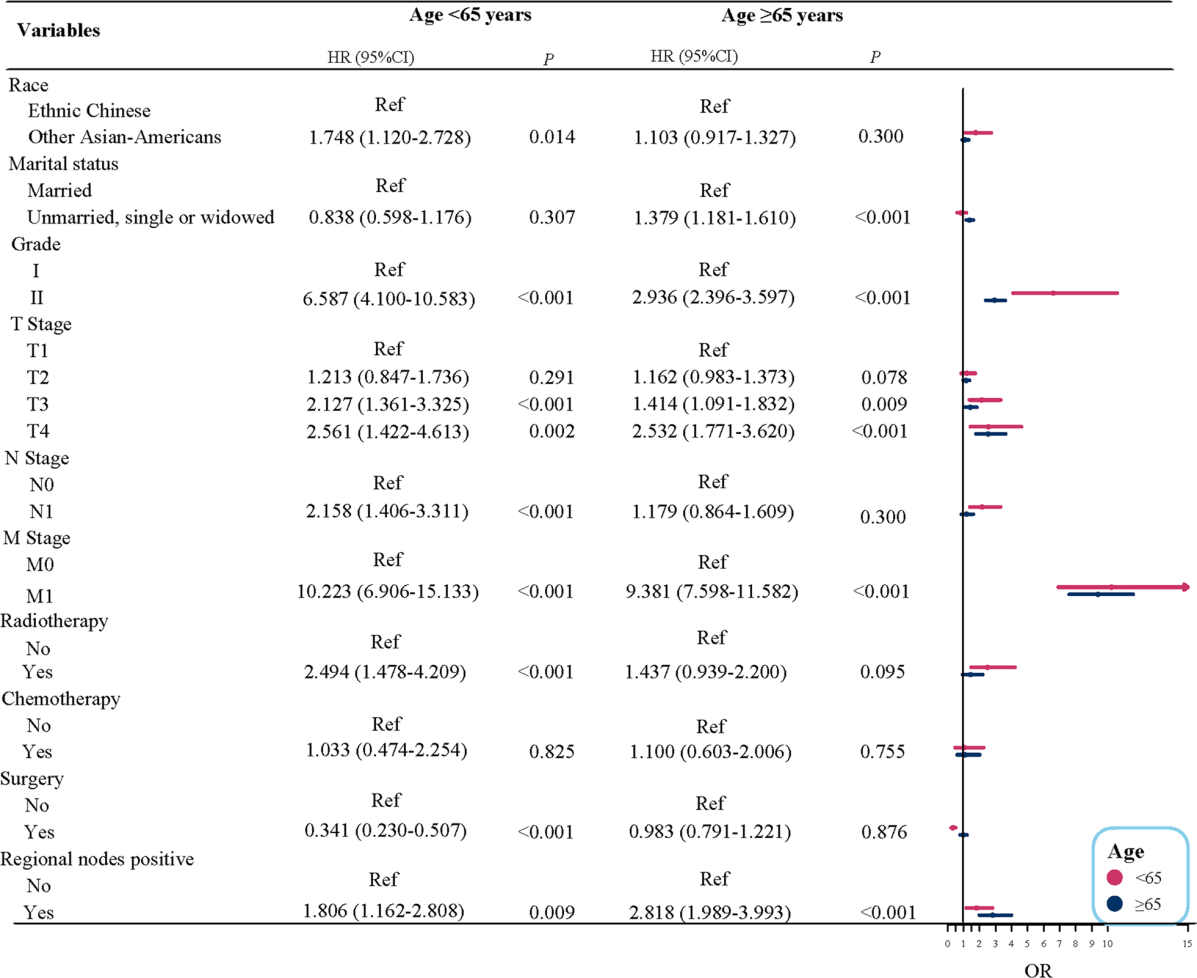
Risk stratification based on age in patients with prostate cancer

Subgroup analysis based on the race for Asian-American PCa patients was conducted. The findings described (Fig. 1) that age ≥ 65 years, grade II, T4 stage, M1 stage, radiotherapy, regional nodes positive had the increased risk of PCa-specific mortality for Ethnic Chinese PCa patients, but among other Asian-American PCa patients, except these factors mentioned above, other marital status (unmarried, single or widowed), T3 stage, N1 stage also had a raised risk of PCa-specific mortality, and while PCa patients with receiving surgical were associated with a reduced risk of PCa-specific mortality. The forest diagram of the race subgroup was revealed in Fig. 1. In addition, we also performed a subgroup analysis for age stratification. Figure 2 suggested that on the basis of presence of competitive risk, other Asian-Americans, grade II, T3 stage, T4 stage, N1 stage, M1 stage, radiotherapy, regional nodes positive were risk factors of PCa-specific mortality among PCa patients younger than 65 years of age. With respect to PCa patients of age ≥ 65, multivariate analysis results displayed that in the presence of competitive risk, other marital status (unmarried, single or widowed), grade II, T3 stage, T4 stage, M1 stage, regional nodes positive were the relevant risk factors of PCa-specific mortality among PCa patients.

## Discussion

PCa, as the one of the most common cancers, has caused the worldwide attention. Despite a significantly decrease in the mortality of PCa patients, the incidence in the United States has still risen in recent years. Significant racial disparities still existed in PCa prognosis among all the United States’ population [21], of which Asian-American men possibly has more advanced stage, higher-grade tumor. The factors of prognosis from PCa among Asian-Americans have got growing focuses, however, there were few studies on PCa survival in the Asian-Americans so far. Nowadays, some studies about survival analysis only discussed the certain outcome, usually ignoring the existence of these competitive events in the analysis, which would lead to biased outcomes [22]. This study aimed to explore the factors of PCa-specific mortality among Asian-American patients with PCa based on the competing-risk model, which is an analytical method to deal with survival data of various potential outcomes. The findings showed that in the presence of competitive risk, age, race, other marital status (unmarried, single or widowed), tumor grade (II), AJCC stage (T3, T4, N1, M1) at diagnosis, radiotherapy, regional nodes positive and surgery were associated with PCa-specific mortality in Asian-Americans PCa patients.

In the present study, age ≥ 65 was identified as a risk factor of the specific death of Asian-American PCa patients, and subgroup analysis of aiming the age manifested that race was no longer a risk factor among Asian-American populations (age ≥ 65), which suggested that elderly patients had higher risk of death than younger groups among all Asian-Americans. Hence, clinicians should pay more attention to PCa patients with aged 65 or older (≥ 65). In our study, other Asian-Americans (such as Japanese, Korean, Filipino, etc.) could increase the risk for PCa patients by 0.220 times compared with Ethnic Chinese, and was an independent risk factor for PCa, which could be related to genetic [23], such as gene mutations and chromosomal, gene, or single nucleotide polymorphisms. Compared with married Asian-Americans patients with PCa, unmarried, single or widowed patients could trigger the higher risk of poor prognosis, which was supported by previous report [24], the reason may be explained that healthy men were more likely to have a beatific married life and married men with a balanced lifestyle could achieve a good prognosis. However, subgroup analysis based on the race showed that other marital status was not statistically significant among Ethnic Chinese population, which may be due to the small sample size of other marital status in this population. Our results also indicated that tumor grade (II) and AJCC stage (T3, T4, N1, M1) at diagnosis were important factors of PCa-specific mortality for Asian-American patients [25, 26]. In general, the higher the tumor grade, the worse the prognosis, as presented in Table 4, Asian-American PCa patients with grade II had an increased risk of death than grade I. At the same time, the results also suggested that the risk of death increased with the increase of T stage. The present results showed that radiotherapy exhibited 0.892 times higher PCa death risk than those without radiotherapy, the reason may be that PCa patients treated with radiotherapy generally were older and had more comorbidities, which affect the probability of a second cancer treatment if disease recurrence [27, 28]. It is worth mentioning that the included Asian-American men patients who had radiotherapy for PCa was less than 4% in our study, which might be not consistent with previous researches [29–31]. Our data was obtained from the SEER database [32]; SEER data are gathered from all clinical settings that diagnose or treat cancer, by trained registrars, and including patients’ demographics, primary tumor site, tumor morphology, stage at diagnosis, and first course of treatment, there may be unavoidable information bias. The included patients in the present study who came from SEER database had some missing information, which may lead to a low radiation treatment data of the current study results. Additionally, just as what M. Raymundo, et al. reported [3], we speculated that Asian-Americans were more likely to choose radical prostatectomy compared to the radiation treatment; nevertheless, these should be cautious in interpreting the results. Future studies should further validate the results of the present study. In addition, our study found that surgery exhibited a lower risk of death among PCa patients [33, 34], simultaneously, the data of subgroup analysis at age manifested that when age was 65 or older, surgery was not considered as a positive factor, it may be related to the following reason: older patients could increase the risk of surgery, they were more likely to be treated with radiotherapy.

The advantages of our study should be pointed. Firstly, the study used a competing-risk model to explore the risk factors of the specific mortality in Asian-American PCa patients, the competing-risk model avoids to overestimate the incidence of outcomes when competing events produced a significant impact. Secondly, variables with clinical value and confounding factors also were selected for subgroup analysis, analyzed in detail the risk factors of death among Asian-American patients with PCa. Nevertheless, there were some limitations in this study, the death risk of Asian-American PCa patients may be associated with other factors (such as diet, exercise, tobacco and wine, etc.), which were not available from the SEER database, large-scale studies are needed to further explore these findings in the future. Besides since the data were designed retrospectively, there may be unavoidable information bias.

## Conclusion

In conclusion, this study aimed to investigate the risk factors of the specific mortality among Asian-American patients with PCa by establishing a competing-risk model based on the SEER database. These findings showed that age, race, marital status, tumor grade (II), AJCC stage (T3, T4, N1, M1) at diagnosis, radiotherapy, regional nodes positive and surgery were associated with the risk of specific mortality among Asian-American PCa patients, and could help clinicians to better evaluate PCa patients’ survival factors and make the clinical personalized decisions and treatment programs.

## Data Availability

The datasets generated and/or analyzed during the current study are available in the SEER repository, https://seer.cancer.gov/.

## Abbreviations

PCa: Prostate cancer
SEER: The Surveillance, Epidemiology, and End Results
HR: Hazard ratio
CI: Confidence interval
AJCC: The American Joint Committee on Cancer
NCI: National Cancer Institute
CIF: Cumulative incidence function
